# Spatial patterns of carbon, biodiversity, deforestation threat, and REDD+ projects in Indonesia

**DOI:** 10.1111/cobi.12500

**Published:** 2015-04-10

**Authors:** Josil P Murray, Richard Grenyer, Sven Wunder, Niels Raes, Julia PG Jones

**Affiliations:** * School of Environment, Natural Resource and Geography (SENRGY), Bangor UniversityLL57 2UW, Bangor, Gwynedd, Wales, United Kingdom; † School of Geography and the Environment, University of OxfordSouth Parks Road, Oxford, OX1 3QY, United Kingdom; ‡ Center for International Forestry Research, Rua do Russel450/601, CEP 22.210–010, Rio de Janeiro, Brazil; § Naturalis Biodiversity CenterDarwinweg 2, P.O. Box 9517, NL-2300 RA Leiden, the Netherlands

**Keywords:** deforestation, ecosystem services, forest degradation, hotspots, protected areas, spatial congruence, áreas protegidas, congruencia espacial, deforestación, degradación del bosque, hotspots, servicios ambientales

## Abstract

There are concerns that Reduced Emissions from Deforestation and forest Degradation (REDD+) may fail to deliver potential biodiversity cobenefits if it is focused on high carbon areas. We explored the spatial overlaps between carbon stocks, biodiversity, projected deforestation threats, and the location of REDD+ projects in Indonesia, a tropical country at the forefront of REDD+ development. For biodiversity, we assembled data on the distribution of terrestrial vertebrates (ranges of amphibians, mammals, birds, reptiles) and plants (species distribution models for 8 families). We then investigated congruence between different measures of biodiversity richness and carbon stocks at the national and subnational scales. Finally, we mapped active REDD+ projects and investigated the carbon density and potential biodiversity richness and modeled deforestation pressures within these forests relative to protected areas and unprotected forests. There was little internal overlap among the different hotspots (richest 10% of cells) of species richness. There was also no consistent spatial congruence between carbon stocks and the biodiversity measures: a weak negative correlation at the national scale masked highly variable and nonlinear relationships island by island. Current REDD+ projects were preferentially located in areas with higher total species richness and threatened species richness but lower carbon densities than protected areas and unprotected forests. Although a quarter of the total area of these REDD+ projects is under relatively high deforestation pressure, the majority of the REDD+ area is not. In Indonesia at least, first-generation REDD+ projects are located where they are likely to deliver biodiversity benefits. However, if REDD+ is to deliver additional gains for climate and biodiversity, projects will need to focus on forests with the highest threat to deforestation, which will have cost implications for future REDD+ implementation.

Los Patrones Espaciales del Carbono, la Biodiversidad, la Amenaza de Deforestación y los Proyectos REDD+ en Indonesia

**Resumen**

Actualmente hay preocupación por que las Emisiones Reducidas de la Deforestación y Degradación del Bosque (REDD+, en inglés) puedan fallar en la entrega de co–beneficios potenciales de la biodiversidad si se enfocan en áreas de alto carbono. Exploramos los traslapes espaciales entre los stocks de carbono, la biodiversidad, las amenazas proyectadas de deforestación y la ubicación de proyectos REDD+ en Indonesia, un país tropical a la vanguardia del desarrollo REDD+. Para la biodiversidad, reunimos datos sobre la distribución de vertebrados terrestres (hábitats de anfibios, mamíferos, aves y reptiles) y plantas (modelos de distribución de especies para ocho familia). Después investigamos la congruencia entre las diferentes medidas de la riqueza de la biodiversidad y los stocks de carbono en la escala nacional y sub–nacional. Finalmente mapeamos proyectos REDD+ activos e investigamos la densidad de carbono y la riqueza potencial de la biodiversidad y modelamos presiones de deforestación dentro de estos bosques en relación con áreas protegidas y no protegidas. Hubo poco traslape interno entre los diferentes hotspots (el 10% más rico de las celdas) de riqueza de especies. Tampoco hubo congruencia espacial consecuente entre los stocks de carbono y las medidas de la biodiversidad: una correlación negativa débil en la escala nacional enmascaró las relaciones altamente variables y no-lineales isla por isla. Los proyectos REDD+ actuales estuvieron ubicados preferencialmente en áreas con una riqueza total de especies y con una riqueza de especies amenazadas más altas pero con densidades de carbono más bajas que en las áreas protegidas y los bosques sin protección. Aunque un cuarto del área total de estos proyectos REDD+ está bajo una presión de deforestación relativamente alta, la mayoría del área REDD+ no lo está. Por lo menos en Indonesia, los proyectos REDD+ de primera generación están ubicados en donde tengan probabilidad de entregar beneficios para la biodiversidad. Sin embargo, si se espera que REDD+ entregue beneficios adicionales para el clima y la biodiversidad, los proyectos necesitarán enfocarse en los bosques con la mayor amenaza de deforestación, lo que tendrá implicaciones de costo para la implementación futura de REDD+.

## Introduction

There has been a lot of interest in the potential of forest carbon sequestration projects such as those being discussed under the climate mechanism to Reduce Emissions from Deforestation and forest Degradation (REDD+) to deliver benefits for biodiversity. Under the proposed mechanism, REDD+ payments are intended to protect threatened tropical forests by providing economic incentives for continued forest integrity (Venter & Koh 2011). The plus in REDD+ expands the scope to include the conservation, sustainable management, and enhancement of forest carbon stocks as means to reduce emissions from deforestation and forest degradation (UNFCCC 2008). Some argue that REDD+ offers “unprecedented” opportunities for biodiversity (Gardner et al. 2012) and provides new funding for conservation (Venter et al. 2009), rehabilitation of critical habitat (Alexander et al. 2011), and the establishment of new protected areas (PAs) (Macdonald et al. 2011). However, many have also drawn attention to potential risks for biodiversity that are associated with preferential targeting of REDD+ projects in high carbon areas, such as displacement of land use pressure (leakage) into high biodiversity but low carbon areas (Harrison & Paoli 2012) and the diversion of funds for forest conservation away from high biodiversity low carbon areas (Phelps et al. 2012).

The degree to which carbon and biodiversity services are colocated in the landscape will influence the potential for delivery of biodiversity benefit; more opportunities are expected where there is congruence between high carbon and biodiversity stocks (Strassburg et al. 2010). There are strong synergies between carbon and biodiversity at the global level (Strassburg et al. 2010). National scale analyses, particularly important for planning REDD+ as an intergovernmental mechanism (Gardner et al. 2012), have been variable in quality and provide ambiguous results. National-level analyses (Madagascar and Bolivia) with finer scale biodiversity data show little congruence between the two or between carbon and biodiversity (Wendland et al. 2010; Sangermano et al. 2012). However, the additional gains from REDD+ for carbon, biodiversity, and other ecosystem services depend on spatially specific threats of deforestation and forest degradation (Busch & Grantham 2013), few, if any, analyses have included both spatial congruence and deforestation threat.

Indonesia is the third largest tropical forest country, a major contributor to global greenhouse gas emissions from deforestation, forest and peat degradation (Margono et al. 2014), and a mega-biodiversity country (Sodhi et al. 2004). Indonesia has made commitments to reduce emissions (GOI 2012) and received significant donor funding for REDD+ implementation (Brockhaus et al. 2012). We assessed the distribution of biodiversity in Indonesia, using species ranges of terrestrial vertebrates (mammals, birds, reptiles, and amphibians) and species distribution models (SDMs) covering 8 plant families which are available for Sundaland only. We explored the congruence between carbon and biodiversity based on 3 measures of richness. We then assessed the location of REDD+ projects relative to deforestation threats and spatially determined potential for these to deliver positive outcomes for carbon and biodiversity.

## Methods

### Data

Our biodiversity analyses were based on recently updated global species range data for the distribution of mammals, reptiles, and amphibians (IUCN 2012), birds (BirdLife International and NatureServe 2012), and SDMs for 8 major plant families (Dipterocarpaceae, Ericaceae, Fagaceae, Lauraceae, Moraceae, Myristicaceae, Sapindaceae, and Leguminosae) in Sundaland. Details on the biodiversity data sets we used are in Supporting Information.

We used newly available high-resolution carbon data sets for above ground biomass (AGB) (Baccini et al. 2012) and soil organic carbon (SOC) up to 100 cm depth (Hiederer & Köchy 2012).

A database of active REDD+ projects in Indonesia was developed for the purpose of this research. We contacted all known REDD+ project developers in Indonesia via email to identify active projects, their central coordinates, and the project size. We achieved a 72% response rate and filled in gaps with best guesses based on available gray literature and Web-based reports. We mapped the location of individual projects based on known project boundaries (*n* = 22), district boundaries for district level projects (*n* = 3), and circular boundaries for projects for which we did not have exact boundary information (*n* = 11). For the circular boundaries, we drew a circle around the project centroid on the diameter of which was based on information about project area provided by project developers. See Supporting Information for details on the REDD+ database.

The PA data set for Indonesia was obtained from the newly updated World Database on PAs (IUCN & UNEP-WCMC 2013). We included PAs in categories I–VI and nationally recognized PAs without an IUCN category (280 in total).

We used the econometric model OSIRIS-Indonesia developed by Busch et al. (2010) to predict deforestation in the absence of REDD+ carbon incentives. The model predicts deforestation based on estimated potential gross agricultural revenues and the cost of converting land from forest to agriculture.

### Analyses

Data sets were analyzed at 5 km × 5 km resolution in the WGS 1984 World Mercator projection. We clipped global data sets to the Indonesian Archipelago (total terrestrial land areas), which covers 79,555 terrestrial cells. (Supporting Information for additional information on the spatial analysis methods.)

Species distribution analyses were based on the polygon vector ranges of 367 amphibian, 281 reptile, 665 mammal, 1559 bird species, and SDMs of 1720 plant species. Following Wang et al. (2013), we calculated species richness as the number of species range polygons that intersect each grid cell. We used 3 measures of species richness: total species, threatened species, and restricted range species.

Threatened species were those classified by the IUCN (2012) as critically endangered, endangered, and vulnerable. Restricted range species were species with a global range in the lowest quartile of their range class (Orme et al. 2005; Grenyer et al. 2006). Species richness of threatened and restricted range species was analyzed only for vertebrates. We identified the richest grid cells (hereafter hotspots [Orme et al. 2005]) for each richness measure for vertebrates and plants (total richness for Sundaland only). We explored the degree to which hotspots overlapped when defined as the richest 10% of cells and the effects of using different hotspot definitions (richest 5%, 10%, 15%, and 25%). We found that regardless of the definition used, there was no overlap between hotspots identified based on different measures of species richness. (Details in Supporting Information.)

Indonesian islands differ in size, isolation, topography, climate, and geology, which results in very different island mean biodiversity and carbon values. We therefore investigated congruence at 3 levels—national and within the 5 major islands (Sumatra, Borneo, Papua, Sulawesi, and Java)—to investigate if national scale patterns are consistent within islands. We selected AGB and SOC up to 100 cm depth based on findings that when congruence was evaluated at 3 soil depths (0, 30, and 100 cm), SOC depth had a clear effect on the congruence patterns, particularly in areas identified as carbon-rich peat swamp forests (see Supporting Information).

Congruence between carbon and the 3 measures of biodiversity richness were assessed using Spearman's rank correlation coefficient; the effective degrees of freedom were corrected by the level of spatial autocorrelation in the data following Dutilleul (1993). We used hexagonal binning (an esthetic mapping technique that shows differences between data-rich and data-sparse parts of the distribution) to visualize the relationship between carbon and biodiversity and fitted a generalized additive model with 95% CIs. All statistical analyses were carried out in R statistical software (R Core Team 2014). Congruence maps were developed in ArcGis 10.1 with the RGB composite band tool.

We assessed the distance and overlap between REDD+ projects (centroid) and PAs (polygon) with the near function in ArcGIS 10.1. We explored the distribution of carbon and biodiversity in Indonesia for 3 categories of forested areas: REDD+ project areas, PAs, and other unprotected forest (outside REDD+ projects and PAs). We defined *forest* as those pixels comprising mangrove, peat swamp forest, lowland forest, lower montane forest, upper montane forest, and plantation or regrowth as in Miettinen et al. (2012). We sampled 1000 random points from all 3 forest categories and compared the means of the 3 groups with analysis of variance followed by a post hoc Tukey's honestly significant difference test to determine categories that were significantly different.

The modeled deforestation data from OSIRIS-Indonesia version 1.5 showing predicted deforestation in the absence of a REDD+ mechanism (Busch et al. 2010) was exported into ArcGIS 10.1 and resampled to 25 km^2^ grid cells (from 9 km^2^). We calculated predicted deforestation per hectare for all grid cells classed as forest in 2010. We extracted predicted deforestation values (percent) for each forested cell and reclassified these into 5 deforestation threat classes (very low to very high) based on natural breaks. Using the zonal statistic function in ArcGIS, we calculated the proportion of REDD+ project area, PAs, and unprotected forests that fell into each deforestation class.

### Limitations

Our analyses relied on available data sets, such as vertebrate vector range maps, which tend to overestimate the likelihood of species occurrence. Some species will be absent in fragments, logged forests, and recently deforested areas. We dealt with this by refining the species range maps and confining our analyses to remaining forest area-based on 2012 forest cover map, as suggested by Jenkins et al. (2013). We also assumed that most species persist in logged or secondary forests based on the large body of literature which supports this (e.g., Sitompul et al. 2013; Struebig et al. 2013; Edwards et al. 2014). Our projected deforestation threat was based on econometric modeling. The results are therefore a scenario-specific prediction of where threats are most likely to occur given the defined model assumptions. The model predicts deforestation based on the conversion of forest land to agriculture.

## Results

### Patterns of Biodiversity Distribution

**Figure 1:**
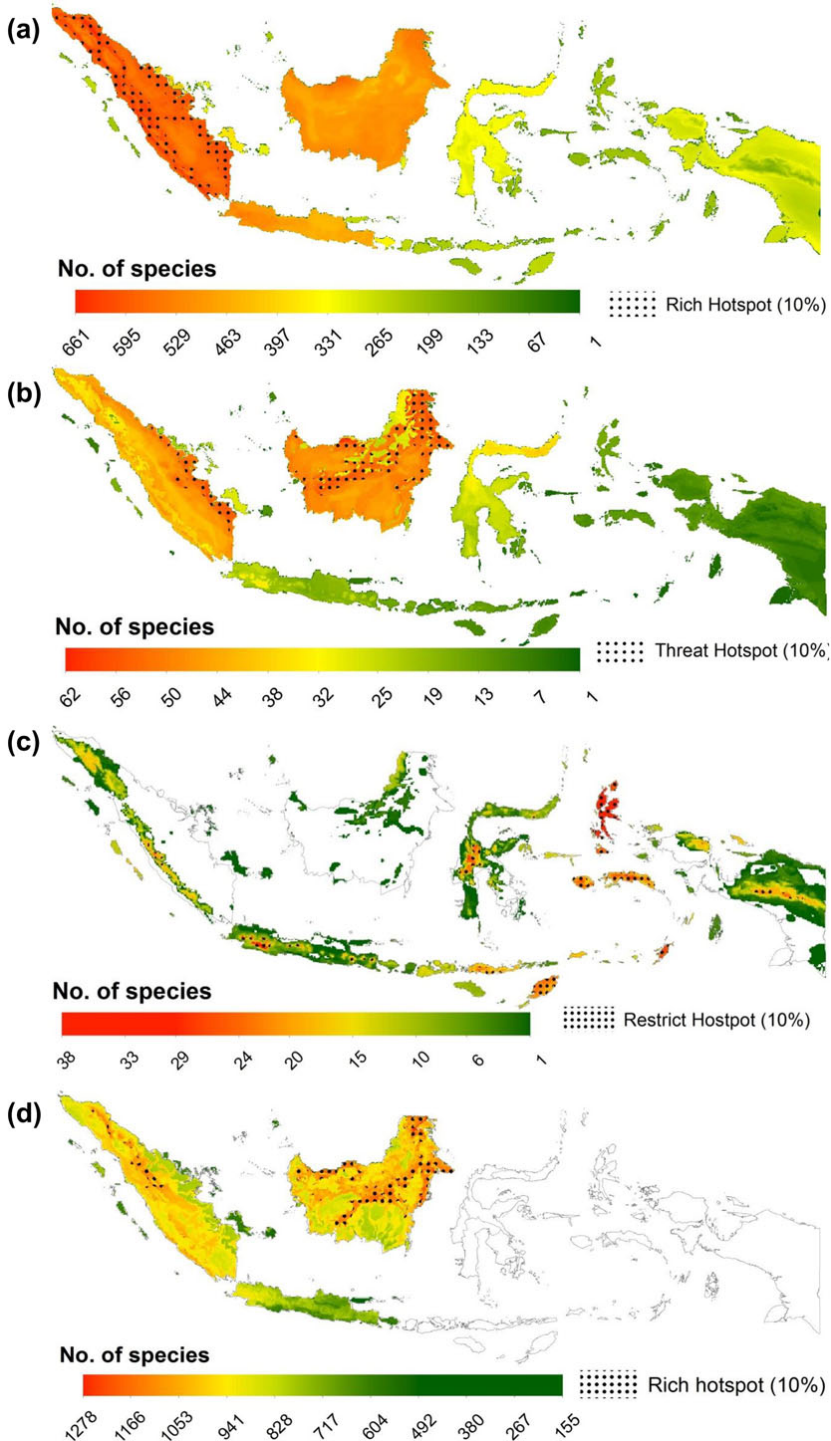
The distribution in Indonesia of (a) total vertebrate species richness, (b) threatened vertebrate species richness, (c) species richness of restricted range vertebrates, and (d) total species richness of vertebrates and plants for Sundaland only, and the location of species rich hotspots (10% of richest cells) for the biodiversity richness measures examined (a–d).

Patterns of potential species richness were highly variable from taxon to taxon and depended strongly on the richness measure used. For total species richness, the highest potential vertebrate species richness was in Sumatra; lower potential species richness was to the East of Wallace's Line in Sulawesi and Papua (Fig. 1a). When both plant and vertebrate data were combined (possible for Sundaland only), the highest total richness shifted from lowland Sumatra to lowland Kalimantan (Fig. 1d), and the northern tip of Kalimantan had the highest total potential species richness (>1270 species in a single cell). Threatened vertebrate species richness was distributed differently. The highest potential richness was concentrated in coastal lowlands of Sumatra and submontane regions of Kalimantan (Fig. 1b), whereas Papua had the lowest potential threatened species richness. Potential restricted range species richness was mostly concentrated in the uplands (Java, Sulawesi, and Papua) and the smaller islands of Buru, Seram, and Halmahera in the Wallacea ecoregion (Fig. 1c). Richness patterns for individual taxa and measures of biodiversity are in Supporting Information.

Hotspots of biodiversity richness identified based on different measures did not generally overlap, further emphasizing that the identification of areas important for biodiversity depended on the measure used (Fig. 1). For example, when hotspots were defined as the richest 10% of cells, no cells were identified as hotspots for all 3 measures of total species richness (vertebrates and plants). Supporting Information contains additional information on the effects of using different hotspot definitions (5%, 10%, 15%, and 25%).

### Congruence between Carbon and Biodiversity

**Figure 2:**
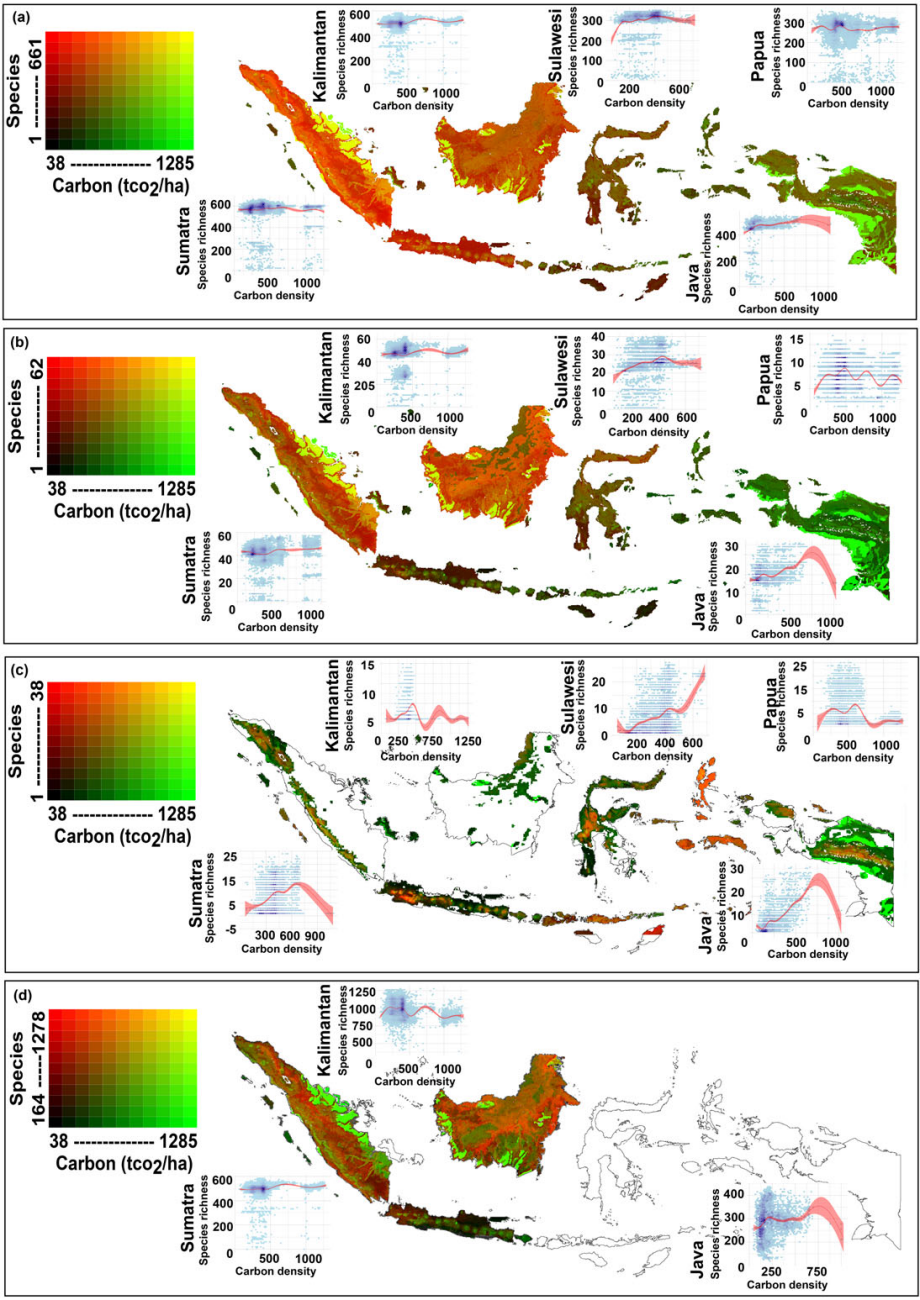
The relationship between biomass carbon (above ground biomass and soil organic carbon) and measures of terrestrial species richness: (a) total vertebrate richness, (b) threatened vertebrate richness, (c) restricted range vertebrate richness, and (d) total vertebrate and plant richness (for Sundaland only) (species, number of species; carbon density units of measure, t CO_2_; 95% CI is displayed around the fitted general additive model; data for island graphs shown on a hexagonal grid shaded logarithmically from white to dark blue to indicate the degree of overplotting).

At the national scale, there was some evidence of a negative relationship between organic carbon stock and all 3 measures of terrestrial vertebrate richness (Table 1, Fig. 2). This negative relationship was significant at the 5% level for threatened species richness and restricted range species richness but was not significant for total species richness. However, this relationship did not hold when analyzed for islands independently (Table 1, Fig. 2).

**Table 1:** Correlations between carbon density (above ground biomass [AGB] and soil organic carbon [SOC] at 100 cm depth) and measures of terrestrial biodiversity richness (total vertebrate richness, threatened vertebrate richness, restricted range vertebrate richness, and total species richness including plants^a^) on 5 islands and all of Indonesia.^b^

	Total richness			Threatened			Restricted			Total richness + plants		
Islands	*r_s_*	*p*	CDF (df)	*r_s_*	*p*	CDF(df)	*r_s_*	*p*	CDF(df)	*r_s_*	*p*	CDF (df)
Kalimantan	0.14	<0.001	1287 (21,028)	0.04	0.159	1418 (21,023)	−0.08	0.016	1021 (3736)	−0.306	<0.001	884 (20,508)
Sumatra	0.01	0.821	519 (17,522)	0.14	0.019	266 (17,480)	0.34	<0.001	1060 (5397)	−0.516	<0.001	860 (16,782)
Java	0.23	<0.001	224 (4832)	0.29	<0.001	162 (4808)	0.61	<0.001	2307 (4224)	0.244	0.007	118 (4639)
Papua	0.00	0.944	446 (15,714)	−0.13	0.114	147 (15,480)	−0.22	<0.001	939 (8858)	–	–	–
Sulawesi	0.22	<0.001	213 (6746)	0.31	0.040	85 (6724)	0.42	<0.001	176 (5165)			
Indonesia	−0.06	0.234	444 (72,684)	−0.08	0.007	1236 (71,996)	−0.06	<0.001	29343 (33,471)	–	–	–

a Only for Sumatra, Kalimantan, and Java.

b Key: *r_s_*, Spearman rank correlation coefficients of all cells; CDF, Clifford's corrected degrees of freedom; df, actual degrees of freedom.

The relationship between carbon density and total species richness was either not significant or only weakly correlated for each of the major islands. With the inclusion of plants, results showed a strong negative relationship between carbon and overall species richness in Kalimantan (*r_s_* = −0.306, *p* < 0.001), and Sumatra (*r_s_* = −0.516, *p* < 0.001) (Table 1, Fig. 2d). This result reflected the fact that peat swamp forests store very large amounts of carbon but do not have particularly high overall plant species richness.

The relationship between carbon density and threatened species richness was neither strong nor monotonic in any of the 4 major islands (Table 1, Fig. 2b). The relationship was strongest in Java, where the correlation was broadly positive (*r_s_* = 0.29, *p* < 0.001). Montane regions of Kalimantan and Papua coincided with the highest concentrations of restricted range vertebrate species (Fig 2c); however, these regions have relatively low carbon densities. Thus, a generally negative relationship between carbon and restricted range species richness was evident in Kalimantan (*r_s_* = −0.075, *p* = 0.016) and Papua (*r_s_* = −0.222, *p* < 0.001) (Table 2, Fig. 2c). The opposite trend was evident on Java (*r_s_* = 0.61, *p* < 0.001), where there was a nearly monotonic positive relationship between carbon and restricted range vertebrate species (Table 2, Fig. 2c), both of which are confined to remaining upland forests. The relationship between each measure of species richness and carbon was also greatly influenced by which taxa were included in the analyses; for example, restricted range birds (*r_s_* = 0.636, *p* < 0.001) and mammals (*r_s_* = 0.49, *p* < 0.001) in Java had strong positive correlation with carbon, whereas plants had a strong negative correlation with carbon in Sumatra (Supporting Information).

**Table 2:** Modeled deforestation in REDD+ project areas, protected areas, and unprotected forests in Indonesia based on 5 deforestation threat categories

		REDD+ areas			Protected areas			Unprotected forest		
Deforestation/ha (%)	Threat level*	Area (1000s ha)	Mean (%)	% of area	Area (1000s ha)	Mean (%)	% of area	Area (1000s ha)	Mean (%)	% of area
0.0002–0.88	Very low	6443	0.3	51	13,193	0.2	71	44,975	0.4	46
1.88–2.13	Low	3280	1.4	26	3408	1.5	18	32,063	1.4	33
2.13–4.55	Medium	2190	2.9	17	1748	2.8	9	15,330	3.0	16
4.55–9.52	High	493	6.1	4	243	5.9	1	3530	6.1	4
9.52–36	Very high	170	12.3	1	53	12.0	0.3	1218	13.1	1

* Deforestation threat category is based on natural breaks, and area (ha) is calculated based on the number of cells that falls within each threat category.

### Carbon, Biodiversity, and Deforestation Threat

We identified 36 active REDD+ projects in 15 provinces of Indonesia (25 projects reported as no longer active). Projects varied in size from site-level activities to those operating at the district or subprovince level. Over half (53%) of the project developers were conservation nongovernmental organizations (NGOs), 33% were private for-profit organizations, and 17% were projects established in collaboration with the Indonesian government or bilateral agencies. At least 25% of REDD+ project centroids overlapped with the boundaries of PAs (Supporting Information).

**Figure 3:**
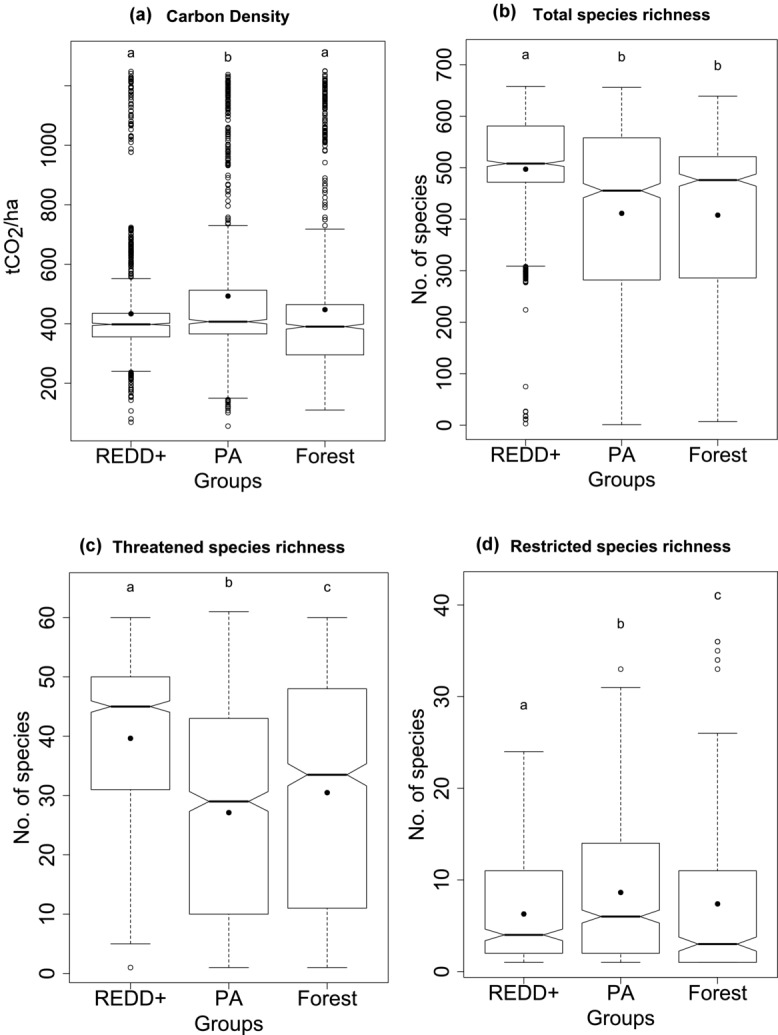
Distribution of carbon and total, threatened, and restricted range vertebrate species richness in REDD+ project areas (REDD+), protected areas (PA), and unprotected forests (Forest) in Indonesia (solid dot, mean; notches in bars, approximate 95% CI around the median value; letters above boxes, different letters show significant difference with Tukey honestly significant difference test). The analysis was of 1000 random sample points from each group.

The REDD+ forests tended to have, on average, lower carbon densities (mean = 433.5 t CO_2_/ha) than PAs (mean = 493.2 t CO_2_/ha) and unprotected forests in Indonesia (mean = 447.6 t CO_2_/ha) (Fig. 3a). Mean carbon density did not differ significantly between REDD+ projects and unprotected forests (*F* = 17.39 on 2877 df, *p* = 3.1 × 10^−8^) (Supporting Information). The REDD+ projects had significantly higher potential total vertebrate species richness (*F* = 130.2 on 2966 df, *p* = 2 × 10^−16^) and threatened species richness (*F* = 152.2 on 2930 df, *p* = 2 × 10^−16^) (Figs. 3b & 3c and Supporting Information). This relationship held true when plants were included in the measures of potential species richness (*F* = 16.35 on 2730 df, *p* = 8.77 × 10^−8^) (Supporting Information). Restricted range species showed a very different pattern; REDD+ projects and unprotected forests had on average lower potential species richness per cell than PAs (*F* = 17.2 on 1631 df, *p* = 4.07 × 10^−8^) (Fig. 3d) (Supporting Information).

At least 23% (or 2.9 million ha) of the area of REDD+ projects was located in forests that had medium to high predicted deforestation threat, whereas 11% (or 2 million ha) of PA and 21% (or 20 million ha) of unprotected forest were under this level of threat. Forests currently not protected by REDD+ or PAs had a much larger area exposed to high deforestation threats; 1 million ha were predicted to be under very high deforestation threat (10–36% deforestation/ha) (Table 2).

## Discussion

### Potential Biodiversity Cobenefits from REDD+

We found that patterns of biodiversity identified depended on the measure of biodiversity used; therefore, the protection of forests with the highest species richness (in Sumatra) may not protect forests with the highest number of threatened species (Kalimantan and coastal Sumatra) or restricted range species (highlands and small islands). Patterns of species richness were also highly variable between taxa, as has been demonstrated globally (Grenyer et al. 2006; Jenkins et al. 2013). Therefore, it is not possible for REDD+ projects to be located in such a way as to be good for all measures of biodiversity simultaneously.

We found no clear and consistent relationship between carbon and any of our proxy measures of biodiversity in Indonesia, there was a weak negative relationship at the national scale, but relationships within islands were sometimes weakly positive, sometimes nonexistent, and sometimes strongly negative. The lack of a clear relationship between carbon and species richness has also been found in South Africa (Egoh et al. 2009) and Madagascar (Wendland et al. 2010). This is perhaps not surprising because of the fundamental ecological differences (definition and substitutability) between carbon and biodiversity (Potts et al. 2013). There are concerns that a lack of congruence between carbon and biodiversity could result in REDD+ investments focusing on high carbon areas which will put biodiversity at risk (Venter et al. 2009; Harrison & Paoli 2012). Although we did not find congruence between carbon stock densities and biodiversity richness in Indonesia, we also did not find REDD+ projects targeting areas with the highest carbon stocks. Instead, they seemed well positioned to deliver biodiversity gains because they tended to be located in areas with higher potential species richness (of total and threatened species).

One factor which may explain why REDD+ projects in Indonesia tended to be located in areas important for biodiversity is that REDD+ development in Indonesia has been spearheaded by conservation NGOs. Such project developers may be seeing REDD+ as a novel funding stream for conservation rather than simply seeking to maximize potential carbon revenues. Our results for Indonesia are consistent with findings from studies in Tanzania (Lin et al. 2014) and Brazil (De Barros et al. 2014), which show evidence of REDD+ initiatives spatially targeting high biodiversity areas. The REDD+ project areas may tend to have lower than average carbon stock because remaining forests outside PAs have mostly been logged (Margono et al. 2014). We also found that many REDD+ projects in our sample are pursuing reforestation and forest restoration as their key project activities, we expect such projects with aims to enhance forest carbon stock to be located in degraded or secondary forests, with perhaps lower than average carbon content.

### Contribution of REDD+ to Conservation in PAs

Implementing REDD+ in PAs has been criticized as not being “additional” (Macdonald et al. 2011) because supposedly PAs are already conserved. However, given the underfunding of many PAs worldwide, it could be argued that improved funding could result in additional gains (Macdonald et al. 2011). Despite their protected status, many PAs in Indonesia are under continuing threat; over 12% of primary forest loss in Indonesia (2000–2012) is located in PAs (Margono et al. 2014), and enforcement is lax (Gaveau et al. 2012). Similarly, we found that PAs were not completely spared from the threat of deforestation; at least 11% (or >2 million ha) of PA area was in areas predicted to have medium to high deforestation threat. We found evidence that REDD+ is indeed being used to support conservation in Indonesia's PAs; at least 25% of REDD+ project boundaries overlapped with PAs (Supporting Information). If REDD+ funding could be used to increase the effectiveness of PAs, the benefits for biodiversity could be large. The REDD+ projects located adjacent to current PAs could also play an important role in softening the matrix, which would reduce the effective isolation of species in the PAs and improve population viability (Jantz et al. 2014).

### Priorities for Achieving Biodiversity Cobenefits with REDD+

Peat swamp forests in Indonesia have global importance in climate mitigation and they are highly threatened because they represent the last frontiers for production of food, pulp, and biofuels (Posa et al. 2011). Recent findings show that 43% (2.6 million ha) of primary forest loss in Indonesia (2000–2012) took place in peatlands, which have an overall increasing rate of loss greater than lowland primary forests (Margono et al. 2014). A large number of REDD+ projects are located in carbon-rich peat swamp forests (Harrison & Paoli 2012). We also found this to be true; however, the total area covered by these projects was much smaller than the area covered by projects on mineral soils (Supporting Information). Highly threatened lowland forests, such as those in the lowlands of Borneo and Sumatra, should remain a priority for future REDD+ planning despite having below-average carbon content. Large expanses of selectively logged forests in Indonesia are now degraded and under high threat of conversion because these are prime agriculture lands where the Indonesian government intends to locate future palm-oil plantations in an attempt to divert palm-oil development away from carbon-rich peat swamp forests and pristine mineral soil forests (Gingold 2010). Margono et al. (2014) found that from 2000 to 2012, 98% (15.8 million ha) of forest loss took place in degraded forests. However, even heavily logged forests can be of high conservation value (Struebig et al. 2013). Meijaard and Sheil (2007) estimate that about 75% of Bornean orangutans (*Pongo pygmaeus*) live in logging concessions, and Sitompul et al. (2013) found that at least 1.6 million ha of Sumatran elephant (*Elephas maximus sumatranus*) habitat is in active logging concessions or in previously logged areas. These forests contain important biodiversity that would be reduced if they were logged again or cleared for oil palm or pulpwood plantations (Edwards et al. 2012). Opportunities for biodiversity in the REDD+ mechanism do not rely on the spatial congruence between carbon and biodiversity alone. The REDD+ policies are important if biodiversity conservation is to be integrated into the national REDD+ architecture (Phelps et al. 2012). Biodiversity-specific management will need to be incorporated in the planning, design, and implementation of REDD+ on the ground (Martin et al. 2013) because protecting existing forest carbon stocks alone will not automatically protect other forest values (Huettner 2012).

### Cost of Delivering Biodiversity Cobenefits in REDD+

Our results show that first-generation REDD+ projects in Indonesia are not necessarily located in the highest threat areas. This is consistent with the findings of Cerbu et al. (2011), who showed that predicted future deforestation appeared to be less of a criteria among first-generation developers for the location of REDD+ projects than the interests of NGOs or government agencies. Early REDD+ projects have built on prior forest management approaches, such as integrated conservation and development projects, as a springboard for REDD+ (Minang & van Noordwijk 2013) and a testing ground for proof of concept (Murdiyarso et al. 2012). The REDD+ projects in our study are in the early stages of development and are operating largely from bilateral REDD+ funding. As the REDD+ mechanism develops, the conditions under which project location is selected will differ; the non-colocation of carbon and biodiversity priority areas in Indonesia highlights an important structural feature which will affect the cost of delivering biodiversity cobenefits in future REDD+ projects.

It can be assumed, based on our findings, that REDD+ projects located in forests most important for biodiversity will cost more per unit of carbon delivered than those located in high carbon forests because forests with the highest biodiversity tend to have low carbon densities but high threat to future deforestation due to high agriculture rent (Busch et al. 2010). Our results show that expanding REDD+ in forest with the lowest deforestation threat (generally on cheaper land) will have low incremental benefits for both biodiversity and carbon. We recommend that future research explicitly assess the costs associated with locating REDD+ projects in forests most important for biodiversity conservation, in light of the limited colocation between carbon and biodiversity we found. A future regulatory mechanism is likely to focus on cost-effective delivery of carbon benefits and not the large-scale delivery of noncarbon benefits (Busch 2013). Biodiversity conservation in the context of REDD+ is therefore likely to require additional investment (Phelps et al. 2012). Options include the introduction of premiums for the delivery of biodiversity benefits (Dinerstein et al. 2013), to allow REDD+ credits to protect forests that are carbon priorities, and use of supplementary funds to protect biodiversity priority areas even when they exhibit low carbon content (Venter et al. 2013). It is an empirical question which of these strategies would be more cost-effective under different contextual preconditions.

We found that patterns of biodiversity varied strongly among taxa and depended on the measure of biodiversity. It would therefore not be possible to place REDD+ projects in areas which are universally good for all measures of biodiversity. In Indonesia carbon stocks correlate poorly with all measures of biodiversity both at the national level and within major islands. However, REDD+ projects under development in Indonesia were located in areas with below-average carbon stock but relatively high biodiversity (according to most measures we used), possibly reflecting the prominent role of conservation NGOs in the development of these first-generation REDD+ projects. Although nearly one-quarter of REDD+ project area was located where deforestation threat was predicted to be relatively high, the majority of REDD+ project area was not in highly threatened forests. This limits the opportunity to achieve the greatest benefits for both emissions reductions and biodiversity conservation. The patterns of biodiversity, threat, and locations of REDD+ projects in Indonesia suggest that biodiversity cobenefits could be achieved through REDD+ in Indonesia, especially if future expansion focused on areas under high deforestation threat. As the world looks toward a global mechanism to address climate change to be agreed upon at the 21st Conference of Parties in Paris at the end of 2015, our findings make an important contribution to debates surrounding the design of REDD+ to maximize the potential for cobenefits. The realized benefits of any REDD+ network will, of course, depend not only on the design and spatial planning but also on the effectiveness of interventions on the ground.

## Supporting Information

Additional information on the biodiversity data set used in the study (Appendix S1), spatial analysis methods (Appendix S2), biodiversity distribution patterns (Appendix S3), effects of using different hotspot definitions and measures of species richness (Appendix S4), and effects of soil organic carbon depth (Appendix S5), the REDD+ project database for Indonesia (Appendix S6), summary statistics for carbon and biodiversity distribution (Appendix S7), and the deforestation model (Appendix S8) are available online. The authors are responsible for the content and functionality of these materials. Queries (other than absence of the material) should be directed to the corresponding authors.
