# Supplementary material for: Spatial patterns of carbon, biodiversity, deforestation threat, and REDD+ projects in Indonesia
Source: Conserv Biol. 2015 Apr 10;29(5):1434–45. doi: 10.1111/cobi.12500 (PMC4654267; doi:10.1111/cobi.12500)
Supplement: Supplementary file 1 [file cobi0029-1434-sd1.docx]

**SUPPORTING INFORMATION**

**Can REDD+ deliver biodiversity benefits? Spatial patterns of carbon, biodiversity and REDD+ projects in Indonesia**

Josil P. Murray, Sven Wunder, Richard Grenyer, Niels Raes and Julia P.G. Jones

Appendix S 1: Additional information on the biodiversity datasets used in the study

We mapped biodiversity richness using improved digital species-range maps (BirdLife International and NatureServe 2012; IUCN 2012, 2013) of well know terrestrial vertebrate taxa (mammals, birds and amphibians and reptiles) (Table S1). These ranges are based on species Extent of Occurrence (EOO) or Area of Occupancy (AOO). Range maps based on EOOs and AOOs are an over estimate of species ranges (Rocchini *et al.* 2011) and are less accurate when compared to species distribution models. However they nonetheless remain an important source of biodiversity data especially when analyzing species richness across large areas (Hurlbert & Jetz 2007). These databases are the most comprehensive available for large study areas like ours and have proven valuable for exploring a variety of broad scale ecological and conservation issues (Jenkins et al. 2013; Cantú-Salazar et al. 2013). These range maps are heavily relied upon when assigning threat status to species (Possingham *et al.* 2002) whereby over 45% of threatened species and 75% of threatened amphibians’ threat status are determined solely based on range measures (Gaston & Fuller 2009). The quality of the IUCN and Birdlife range maps have improved in recent years (Jenkins *et al.* 2013) and we consider their use is here is justified. We included plants as a proxy for biodiversity with modelled data made available by (Raes *et al.* 2009, 2013) which covers Sundaland^[[1]](#footnote-1)^ i.e. three out of the five major islands in our study area (Java, Sumatra, Kalimantan and Sulawesi). The eight modelled plant families are highly diverse and have characteristic roles in all ecosystems ranging from tropical lowland to montane forests.

## **Table S 1: Biodiversity dataset for Indonesia covering five taxonomic groups for three measures of biodiversity richness.**

| **Taxonomic groups** | **Species richness measure (number of species)** | | |
| --- | --- | --- | --- |
|  | **Overall species** | **Threatened species** | **Restricted range species** |
| **Amphibians** | 369 | 28 | 81 |
| **Mammals** | 668 | 162 | 178 |
| **Birds** | 1559 | 122 | 339 |
| **Reptiles** | 281 | 6 | 34 |
| **Plants*** | 1720 | - | - |
| **Total** | 2877 | 318 | 632 |

Appendix S 2: Additional information on spatial analysis methods

**Projection:** We chose the WGS84 World Mercator as the coordinate system because we needed one that is zone independent and preferably in meters for ease of further analysis. The Mercator projection is centered around the equator, causing minimal distortion in the distance and size.

**Spatial resolution:** To test for the effect of the scale of analysis on our result we assessed the congruence between carbon and three measures of biodiversity two spatial resolutions - 5km x 5km and 1km x 1km for central Kalimantan. The results showed that there were no differences in the correlation co-efficient. The effective sample size (corrected degrees of freedom) also generally did not differ. The results of this sensitivity analysis indicate that analyzing the data at a finer spatial resolution of 1km x 1km does not change the results. Figure S1 (below) shows the spatial relationship between carbon and biodiversity at both resolutions; Table S2 (below) shows the results for the correlation analysis.

While the sensitivity analysis shows that there is no difference in the correlation between carbon and biodiversity measures at the different resolution, we are aware of the pitfalls of using coarse resolution for our analysis, however our choice of resolution was based on a combination of factors, here we justify why we chose to carry out our analysis at 5km x 5km resolution:

a. It is usually the case when using best available datasets for analysis on large areas that datasets will be at different spatial resolutions. The dataset we used with the finest resolution is the carbon density data (500 m x 500 m) and the coarsest data is our plant SDM dataset is 10km x 10km resolution. Our choice of 5km x 5km was a compromise in order to get a balance between all our datasets.

b. Global species range maps like the IUCN species range maps we use in our analysis were not designed to be used at such fine resolution (Jenkins et al. 2013). Bringing the resolution down to 1km will not necessarily increase the accuracy. In spite of this limitation, the IUCN range maps are currently still the best data available for assessing very large areas for large numbers of taxa like ours. As for our plant SDMs, the spatial resolution of approximately 10 × 10 km at the equator is considered the accuracy range in which they can carry out their analysis (Raes et al. 2013).

c. Lastly, the speed of analysis was definitely a factor especially if increasing the resolution does not increase the accuracy. Carrying out such fine scale analysis for the whole of Indonesia and individual islands would take a very long time to run. For example even when restricting the sensitivity test to just Central Kalimantan, it took approximately 4 whole days to complete the analysis for 1km x 1km resolution (Table 1B). Running at this scale for the whole of Indonesia and islands separately would have required computing power we don’t have available.

Table S 2: Spatially restricted correlations test between carbon densities three measures of terrestrial biodiversity richness - a) total vertebrate richness, b) threatened vertebrate richness c) restricted range vertebrate richness and d) total species. Note: rs is Spearman rank correlation coefficients of all cells, CDF is Clifford’s corrected degrees of freedom and df is the actual degrees of freedom.

| **Central Kalimantan: Congruence between carbon (AGB + SOC at 100cm depth) and three measureas of biodiversity richness at two spatial resolutions** | | | | | | | | | | | | |
| --- | --- | --- | --- | --- | --- | --- | --- | --- | --- | --- | --- | --- |
| **Spatial Resolution** | **Measures of vertebrate species richness** | | | | | | | | | **d. Total Richness (+ Plants)** | | |
|  | **a. Total Richness** | | | **b. Threatened** | | | **c. Restricted** | | |  |  |  |
|  | **Rs** | **P-value** | **CDF (df)** | **Rs** | **P-value** | **CDF(df)** | **Rs** | **P-value** | **CDF(df)** | **Rs** | **P-value** | **CDF (df)** |
| **5km x 5km** | 0.20 | 0.032 | 113 (6112) | 0.05 | 0.493 | 212 (6112) | -0.02 | 0.699 | 302  (457) | -0.35 | 0.001 | 82 (6078) |
| **1km x 1km** | 0.20 | 0.009 | 168 (152824) | 0.05 | 0.403 | 288 (152824) | -0.02 | 0.403 | 1408 (11449) | -0.35 | <0.001 | 138(151974) |


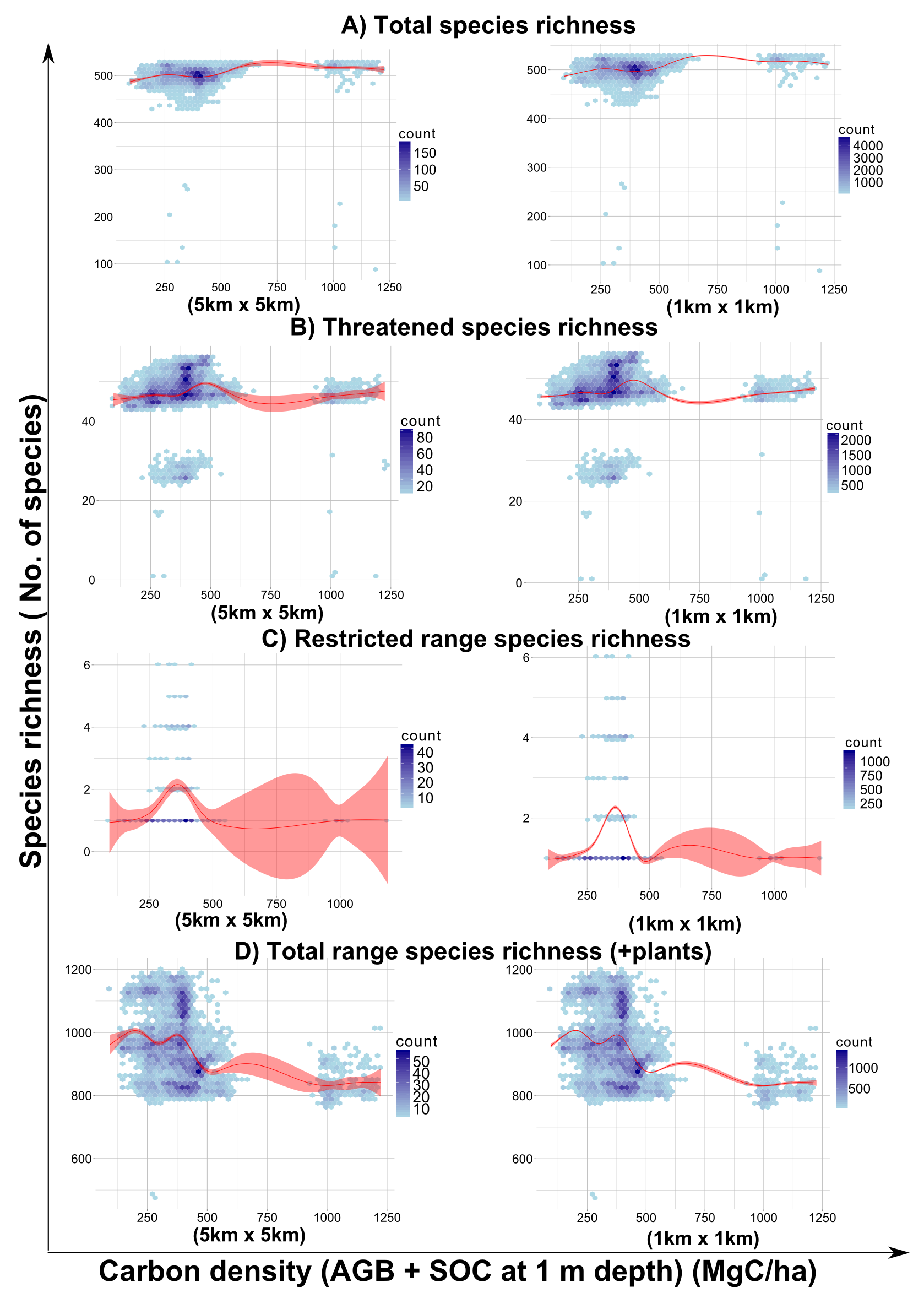


Figure S 1: Spatial relationship between carbon ( Above Ground Biomass – ABG and Soil organic Carbon – SOC) at 1 meter depth and three measures of vertebrate richness including total vertebrate (A – C) and plant richness (D) at 5km x 5km and 1km x 1km resolution.

Appendix S 3: Additional information on biodiversity distribution patterns

The overall pattern of biodiversity distribution is a reflection of the patterns in the various taxa which make up the dataset. All taxa were treated equally i.e. no weighting was carried out in our analysis. Birds dominate the patterns total richness and threatened species richness while restricted range mammals are numerically more abundant and these dominate the richness pattern for restricted range species (Fig. S1). Plants have a distinct richness distribution pattern from vertebrates and therefore significantly change the pattern of total richness when they are included. Figure S1 shows how species richness varies between taxa based on different measures of richness.

Figure S 2: Taxon level species distribution according to three measures of biodiversity.


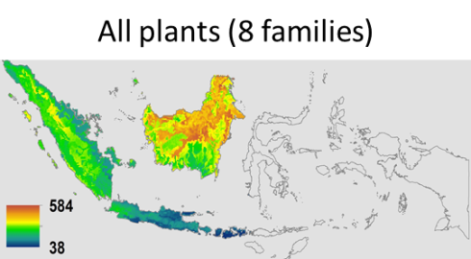

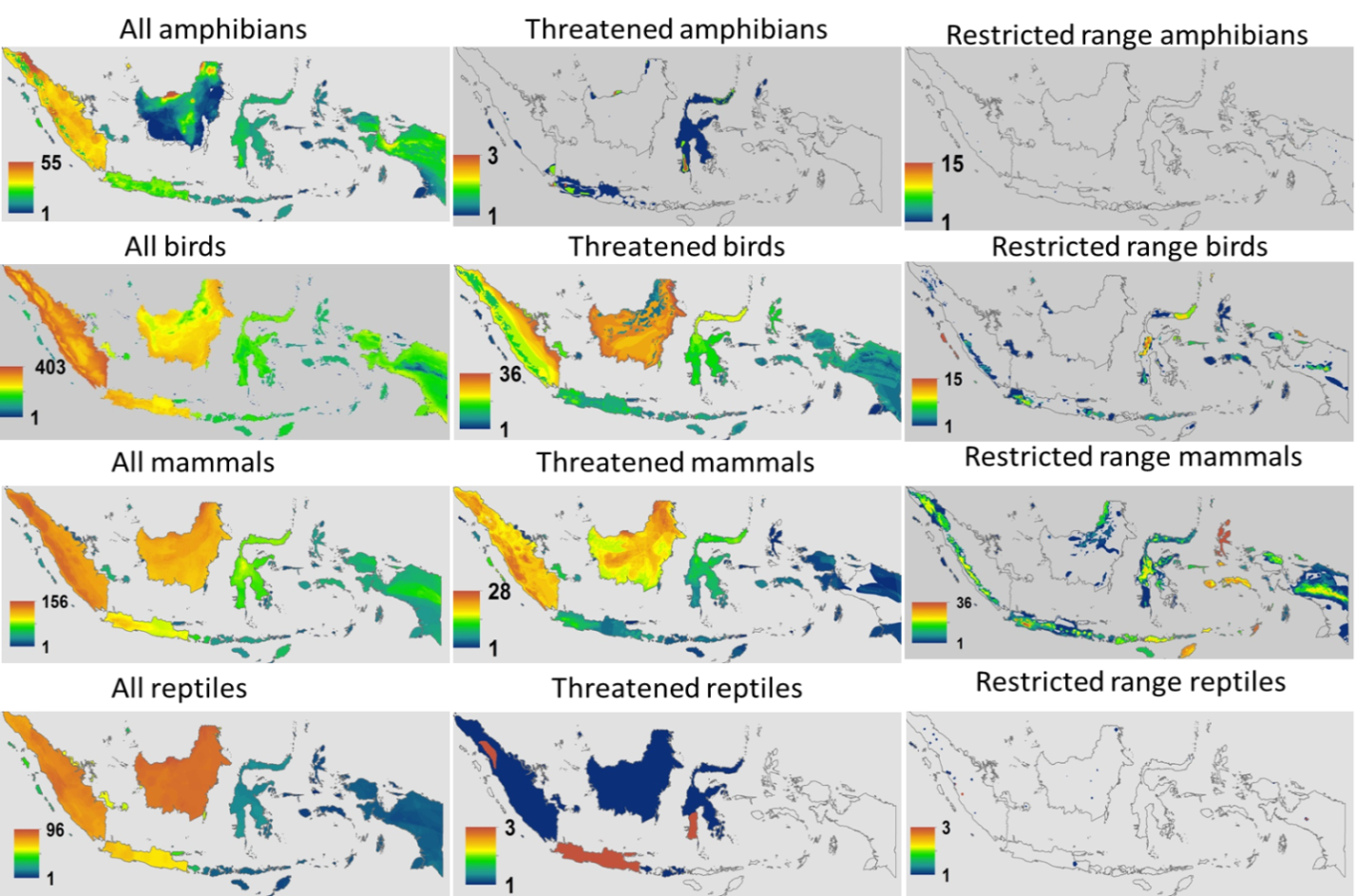


Appendix S 4: Additional information on the effects of using different hotspot definitions and different measures of species richness

Areas identified as important for biodiversity (i.e. hotspots) in Indonesia vary strongly depending on the measure used. The three hotspots occupied 23.8% (18969 cells) of Indonesian land surface but only 2% (1602 cells) had an overlap of at least two hotspots (Table S3).

We also found that that this overlap generally does not change with change of “hotspot definition”. Even when defining hotspots as the richest 25% of cells, there is still very little overlap between hotspots of the three measures of biodiversity used in this study.

Regardless of the hotspot definition used, Kalimantan is a priority area for total (vertebrate + plants) species richness; Sumatra is a priority for vertebrate species richness, while the smaller islands and mountains of Papua are hotspots for restricted range species. With the inclusion of plants, the coastal and sub-montane forests of Sumatra lose their role as centres of total species richness while sub-montane areas in Kalimantan show prominence (Fig. S2).

Table S 3: Degree of overlap between hotspots for different measures of biodiversity (overall species, threatened species and restricted range species richness) overlap with different hotspot definitions.

| **Hotspot Overlaps** | **Hotspot definitions** | | | | | | | | |
| --- | --- | --- | --- | --- | --- | --- | --- | --- | --- |
|  | **5%** | **10%** | **15%** | **25%** |  | **5%** | **10%** | **15%** | **25%** |
|  | **Vertebrate** | | | |  | **Vertebrate (+ plants)** | | | |
| 1 | 9767 | 17367 | 27419 | 31456 |  | 5890 | 9546 | 13707 | 20467 |
| 2 | 491 | 1602 | 5662 | 9081 |  | 654 | 1909 | 3681 | 6969 |
| 3 | 0 | 0 | 2 | 12 |  | 0 | 0 | 0 | 1 |
| Total cells | 10258 | 18969 | 33083 | 40549 |  | 6544 | 11455 | 17388 | 27437 |
| % of Indonesia | 12.9 | 23.8 | 41.6 | 51.0 |  | 8.2 | 25.5 | 38.6 | 61.0 |

Figure S 3: Hotspots of species richness based on four different hotspot definition and according to three different measures of species richness.

**
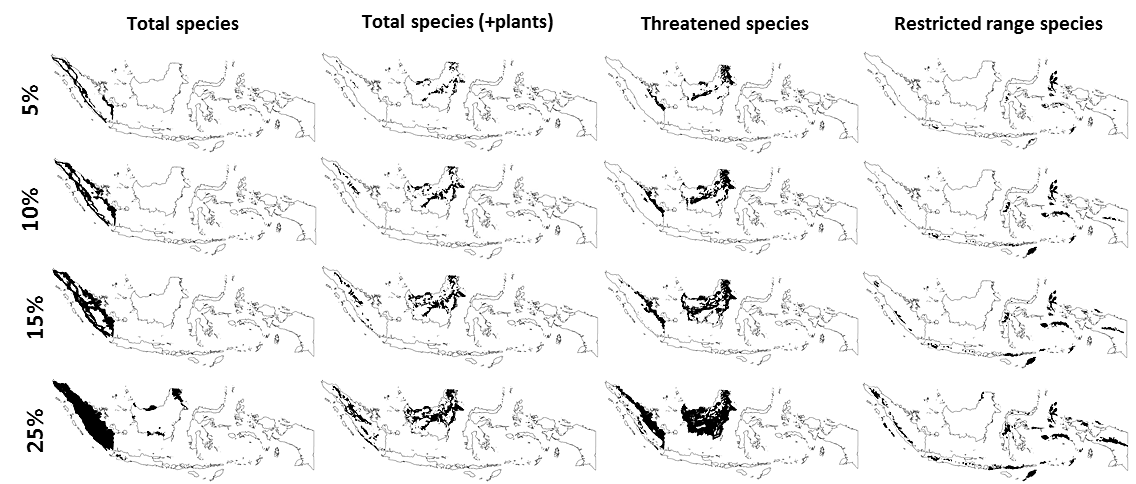
**

Table S 4: Correlation between carbon and biodiversity at the taxa level according to 4 major islands in Indonesia and three measures of species richness.

| **Major Islands** | **Birds** | | | | | | | | |
| --- | --- | --- | --- | --- | --- | --- | --- | --- | --- |
|  | **Total species** | | | **Threatened sp.** | | | **Restricted range sp.** | | |
|  | **Rs** | **P-value** | **CDF (df)** | **Rs** | **P-value** | **CDF (df)** | **Rs** | **P-value** | **CDF (df)** |
| **Kalimantan** | 0.194 | <0.001 | 384(21097) | 0.051 | 0.173 | 712(21083) | -0.053 | 0.121 | 845(3465) |
| **Sumatra** | 0.097 | 0.05 | 408(17555) | 0.035 | 0.681 | 140(17549) | 0.387 | 0 | 2096(5071) |
| **Java** | 0.283 | <0.001 | 206(4924) | 0.29 | 0 | 176(4919) | 0.636 | 0 | 1178(4108) |
| **Papua** | 0.139 | 0.028 | 248(15746) | -0.013 | 0.861 | 173(15685) | -0.196 | 0 | 1164(8548) |
| **Major Islands** | **Amphibians** | | | | | | | | |
|  | **Total species** | | | **Threatened sp.** | | | **Restricted range sp.** | | |
|  | **Rs** | **P-value** | **CDF (df)** | **Rs** | **P-value** | **CDF (df)** | **Rs** | **P-value** | **CDF (df)** |
| **Kalimantan** | 0.056 | 0.052 | 1185(19452) | -0.111 | 0.2 | 132(497) | -0.064 | 0.11 | 620 (3) |
| **Sumatra** | -0.064 | 0.275 | 291(17536) | -0.426 | 0.001 | 61(678) | -0.06 | 0.726 | 34 (35) |
| **Java** | -0.125 | 0.052 | 238.162 (4912) | 0.306 | <0.001 | 2267 (3243) | - | - | - |
| **Papua** | -0.146 | 0.056 | 170(15664) | None | | | 0.015 | 0.884 | 100 (109) |
| **Major Islands** | **Mammals** | | | | | | | | |
|  | **Total species** | | | **Threatened sp.** | | | **Restricted range sp.** | | |
|  | **Rs** | **P-value** | **CDF (df)** | **Rs** | **P-value** | **CDF (df)** | **Rs** | **P-value** | **CDF (df)** |
| **Kalimantan** | −0.015 | 0.664 | 822(21077) | −0.029 | 0.574 | 369(21057) | -0.091 | 0.203 | 194 (265) |
| **Sumatra** | −0.185 | 0.034 | 130(17530) | −0.181 | 0.122 | 72(17509) | −0.099 | 0.009 | 693(2061) |
| **Java** | 0.286 | <0.001 | 181(4904) | 0.353 | <0.001 | 143(4909) | 0.49 | <0.001 | 1252(1749) |
| **Papua** | −0.299 | <0.001 | 148(15669) | -0.202 | <0.001 | 564 (10011) | −0.285 | <0.001 | 236(2052) |
| **Major Islands** | **Reptiles** | | | | | | | | |
|  | **Total species** | | | **Threatened sp.** | | | **Restricted range sp.** | | |
|  | **Rs** | **P-value** | **CDF (df)** | **Rs** | **P-value** | **CDF (df)** | **Rs** | **P-value** | **CDF (df)** |
| **Kalimantan** | 0.136 | <0.001 | 1287 (21028) | 0.037 | 0.159 | 1418 (21023) | -0.075 | 0.016 | 1021 (3736) |
| **Sumatra** | 0.01 | 0.821 | 519 (17522) | 0.143 | 0.019 | 267 (17480) | 0.336 | <0.001 | 1060 (5397) |
| **Java** | 0.23 | <0.001 | 224 (4832) | 0.293 | <0.001 | 162 (4808) | 0.61 | <0.001 | 2307 (4224) |
| **Papua** | 0.003 | 0.944 | 446 (15714) | -0.13 | 0.114 | 147 (15480) | -0.222 | <0.001 | 939 (8858) |
| **Major Islands** | **Plants** | | | | | | | | |
|  | **Total species** | | | **Threatened sp.** | | | **Restricted range sp.** | | |
|  | **Rs** | **P-value** | **CDF (df)** | **Rs** | **P-value** | **CDF (df)** | **Rs** | **P-value** | **CDF (df)** |
| **Kalimantan** | -0.374 | <0.001 | 1287 (21028) |  |  |  |  |  |  |
| **Sumatra** | -0.634 | <0.001 | 519 (17522) |  |  |  |  |  |  |
| **Java** | 0.157 | 0.075 | 224 (4832) |  |  |  |  |  |  |
| **Papua** |  |  |  |  |  |  |  |  |  |
| **CDF = Corrected Degrees of Freedom** | | | |  |  |  |  |  |  |
| **df = actual degrees of freedom** | | |  |  |  |  |  |  |  |
| **Rs = Correlation Coefficient** | | |  |  |  |  |  |  |  |

Appendix S 5: Additional information on congruence patterns and effects of soil organic carbon depth

We found a number of factors influencing the congruence between carbon and biodiversity in Indonesia. Firstly, we found that the correlations between the two variables change when being analyzed for Indonesia as a whole or when analyzed by island. Secondly, we also found big changes in the congruence patterns when we considered soil organic carbon (or not) as our measure of carbon stock. We tested this by running the congruence analysis under three carbon scenarios i) AGB (Above-ground biomass only) ii) AGB+SOC30 (Above-ground biomass + soil organic carbon at 30cm depth) and ii) AGB + SOC 100cm (results presented in the main findings). Statistically, there are changes in the correlation between carbon and biodiversity when comparing AGB only and AGB + SOC100cm but less so for AGB+SOC30cm. In Java where SOC depth does not affect overall carbon stock, we also find that the correlation coefficient does not change for all measure of richness and at all SOC depths. Clear differences can be seen for threatened species across the three SOC depths especially in Kalimantan, Papua and Sumatra. We found little change with SOC depth for restricted range species perhaps due to the fact that restricted range species are concentrated on montane areas which are not affected by change in SOC depth. We can link the differences in congruence patterns with the change in soil depth to the effect of high carbon peat swamp forests. This is clearer when visualised in Fig.S4 where clusters of cells with very high changes the over GLM. Because of the importance of peat swamp forests in both climate mitigation and biodiversity conservation in Indonesia, we therefore include SOC up to 100 cm depth in the main analysis. Peat swamp forests in Indonesia are responsible for 61% of carbon emission in Indonesia (Rehman et al 2014) and the most common deforestation pressure in carbon rich peat swamp forests is the conversion to oil palm plantations (>70% between 1990s – 2000) (Ramdani & Hino 2013).


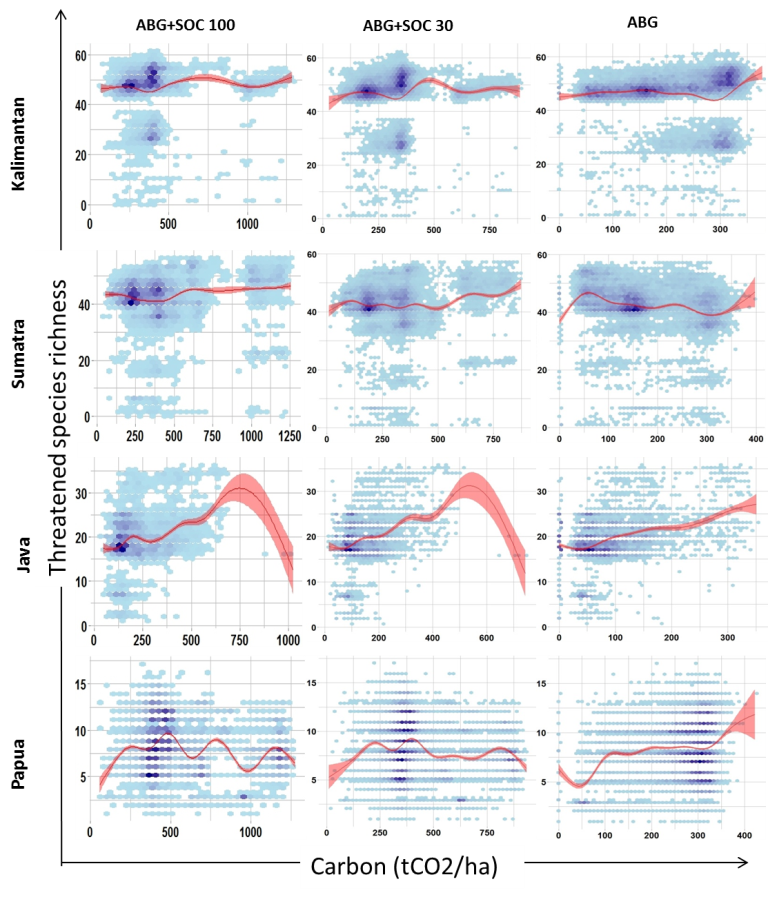


Figure S 4: Using correlation between threatened species richness as an example, the graphs show how carbon and threatened species interact across 4 major islands in Indonesia with visible clusters of carbon rich cells in Kalimantan and Sumatra at ABG+SOC

Table S 5: Effects of soil organic carbon (SOC) depth on congruence patterns between carbon and biodiversity in Indonesia with A) Above Ground Biomass (AGB) and SOC at 30 cm depth and B) AGB only

| **Congruence between carbon (AGB + SOC at 100cm depth) and three measureas of biodiversity richness** | | | | | | | | | | | | | |
| --- | --- | --- | --- | --- | --- | --- | --- | --- | --- | --- | --- | --- | --- |
| **Major Islands** | **Measures of vertebrate species richness** | | | | | | | | | | **d. Total Richness (+ Plants)** | | |
|  | **a. Total Richness** | | | **b. Threatened** | | | **c. Restricted** | | | |  |  |  |
|  | **Rs** | **P-value** | **CDF (df)** | **Rs** | **P-value** | **CDF(df)** | **Rs** | **P-value** | **CDF(df)** | **Rs** | | **P-value** | **CDF (df)** |
| **Kalimantan** | 0.14 | <0.001 | 1287 (21028) | 0.04 | 0.159 | 1418 (21023) | -0.08 | 0.016 | 1021 (3736) | −0.306 | | <0.001 | 884 (20508) |
| **Sumatra** | 0.01 | 0.821 | 519 (17522) | 0.14 | 0.019 | 266 (17480) | 0.34 | <0.001 | 1060 (5397) | −0.516 | | <0.001 | 860 (16782) |
| **Java** | 0.23 | <0.001 | 224 (4832) | 0.29 | <0.001 | 162 (4808) | 0.61 | <0.001 | 2307 (4224) | 0.244 | | 0.007 | 118 (4639) |
| **Papua** | 0.00 | 0.944 | 446 (15714) | -0.13 | 0.114 | 147 (15480) | -0.22 | <0.001 | 939 (8858) | - | | - | - |
| **Sulawesi** | 0.22 | <0.001 | 213(6746) | 0.31 | 0.040 | 85(6724) | 0.42 | <0.001 | 176(5165) |  | |  |  |
| **Indonesia** | -0.06 | 0.234 | 444 (72684) | -0.08 | 0.007 | 1236 (71996) | -0.06 | <0.001 | 29343 (33471) | - | | - | - |
| **Congruence between carbon (AGB + SOC at 30cm depth) and three measureas of biodiversity richness** | | | | | | | | | | | | | |
| **Major Islands** | **Measures of vertebrate species richness** | | | | | | | | | | **d. Total Richness (+ Plants)** | | |
|  | **a. Total Richness** | | | **b. Threatened** | | | **c. Restricted** | | | |  |  |  |
|  | **Rs** | **P-value** | **CDF (df)** | **Rs** | **P-value** | **CDF(df)** | **Rs** | **P-value** | **CDF(df)** | **Rs** | | **P-value** | **CDF (df)** |
| **Kalimantan** | 0.11 | 0.002 | 776(21047) | 0.02 | 0.395 | 1304(21035) | -0.01 | 0.898 | 652(3746) | -0.21 | | <0.001 | 799(20369) |
| **Sumatra** | 0.02 | 0.666 | 410(17463) | 0.11 | 0.104 | 237(17444) | 0.36 | <0.001 | 1122(5391) | -0.46 | | <0.001 | 1124(16666) |
| **Java** | 0.27 | <0.001 | 217(4899) | 0.36 | <0.001 | 217(4899) | 0.65 | <0.001 | 2088(4286) | 0.149 | | <0.001 | 215(4662) |
| **Papua** | 0.028 | 0.478 | 629(15735) | -0.11 | 0.221 | 119(15508) | -0.27 | <0.001 | 975(8866) | - | | - | - |
| **Sulawesi** | 0.240 | <0.001 | 201(6746) | 0.33 | 0.004 | 74(6724) | 0.40 | <0.001 | 178(5165) |  | |  |  |
| **Indonesia** | -0.11 | 0.048 | 24(72684) | -0.11 | <0.001 | 985(71996) | -0.05 | <0.001 | 28186(33471) | - | | - | - |
| **Congruence between carbon (AGB only) and three measureas of biodiversity richness** | | | | | | | | | | | | | |
| **Major Islands** | **Measures of vertebrate species richness** | | | | | | | | | | **d. Total Richness (+ Plants)** | | |
|  | **a. Total Richness** | | | **b. Threatened** | | | **c. Restricted** | | | |  |  |  |
|  | **Rs** | **P-value** | **CDF (df)** | **Rs** | **P-value** | **CDF(df)** | **Rs** | **P-value** | **CDF(df)** | **Rs** | | **P-value** | **CDF (df)** |
| **Kalimantan** | -0.03 | 0.547 | 355 (21131) | -0.04 | 0.465 | 341 (21119) | 0.27 | 0.002 | 124 (3782) | 0.19 | | 0.02 | 151(20398) |
| **Sumatra** | 0.02 | 0.604 | 923(17623) | -0.23 | <0.001 | 401(17581) | 0.35 | <0.001 | 827(5463) | -0.21 | | <0.001 | 799(20369) |
| **Java** | 0.28 | <0.001 | 148(5181) | 0.37 | <0.001 | 153(5169) | 0.59 | <0.001 | 2782(4551) | 0.05 | | 0.400 | 273(4894) |
| **Papua** | 0.16 | <0.001 | 684(15955) | 0.21 | 0.059 | 77(15679) | -0.25 | <0.001 | 2548(8921) | - | | - | - |
| **Sulawesi** | 0.23 | <0.001 | 309(6903) | 0.31 | 0.005 | 77(6874) | 0.35 | <0.001 | 298(5260) | - | | - | - |
| **Indonesia** | -0.15 | 0.003 | 399(74505) | -0.10 | 0.138 | 222(73649) | 0.11 | <0.001 | 7715(34432) | - | | - | - |
|  |  |  |  |  |  |  |  |  |  |  | |  |  |
| **CDF = Corrected Degrees of Freedom** | | | |  |  |  |  |  |  |  | |  |  |
| **df = actual degrees of freedom** | | | |  |  |  |  |  |  |  | |  |  |
| **Rs = Correlation Coefficient** | | |  |  |  |  |  |  |  |  | |  |  |
| **AGB - Above Ground Biomass , SOC = Soil organic carbon** | | | | | |  |  |  |  | |  |  |  |

Appendix S 6: Additional information about the REDD+ project database for Indonesia

We developed a preliminary list of REDD+ projects in Indonesia by cross-referencing eight online databases^[[2]](#footnote-2)^ and two recently published reports on REDD+ projects in Indonesia (Pusat Standardisasi dan Lingkungan 2011; Mardiastuti 2012). We contacted all project developers identified and asked i) if their project is still active, ii) the project’s central coordinates, and iii) the project’s size. Projects not considered as REDD+ projects by their developers were not included in the analysis. We achieved a 72% response rate. For non-respondents, project information was obtained from online websites, reports and project design documents. A total of 36 active REDD+ projects were identified. We mapped the location of individual projects using known project boundaries (n = 22), district boundary for district level projects (n = 3) and circular boundaries for projects we do not have boundary information (n = 11). Circular boundaries were determined by drawing a circular buffer around project centroid using information about project area size provided by project developers. The protected area dataset for Indonesia was obtained from the newly updated World Database on Protected Areas. Table S7 below shows the REDD+ project area (ha)which fall within forest land use categories (as defined in this paper). We found that approximately half of the REDD+ project area is located in lowland forests (44.2%). Like Paoli et al. (2010), we found that many of these REDD+ projects had parts of their boundary on peatlands however in terms of area covered, it is small (9%). REDD+ projects on mineral soil forests are also relatively bigger than those on peatlands. We also found that at least 23 % of REDD+ project area is located in plantation or regrowth areas which includes degraded forests such as dense shrublands and young secondary forests and intensively logged forests where canopy cover is dominated by regrowth (Miettinen *et al.* 2012).

Table S 6: List of active REDD+ projects in Indonesia, their project developers and organization type. Note: Data updated July 2013. Organization type code: 1 = NGO, 2 = Bilateral/ Government and 3 = Private)

| **ID** | **Project** | **Project Proponent** | Code |
| --- | --- | --- | --- |
| 1 | Ulu Masen Ecosystem – A Triple-Benefit Project | Carbon Conservation | 3 |
| 2 | Leuser Ecosystem REDD Project | Global EcoRescue | 3 |
| 3 | Batang Toru REDD project | Conservation International | 1 |
| 4 | Tesso Nilo Pilot Project | World Wide Fund for Nature (WWF) | 1 |
| 5 | The Kampar Peninsular REDD+ Project | Sinarmas Forestry & PT Putra Riau Perkasa | 3 |
| 6 | Kampar Ring - REDD and HTI | Asia Pacific Resources International Limited (APRIL) | 3 |
| 7 | REDD+ Pilot Project in Bengkalis and Siak | Sinarmas Forestry | 3 |
| 8 | Siberut Project | Global Green | 3 |
| 9 | Berbak Carbon Inititiative (BCI) | Zoological Society London (ZSL) | 1 |
| 10 | Community Carbon Pool in Jambi: Kerinci Seblats buffer zone forest | Flora and Fauna International (FFI) | 1 |
| 11 | Rehabilitation of Degraded Peatland in Ogan Komering Ilir, South Sumatra | Sinarmas Forestry | 3 |
| 12 | Lebong Carbon Conservation Project | Arthasuaka & Carbon Conservation | 3 |
| 13 | Meru Betiri National Park | Forest Research & Development Agency (FORDA) | 2 |
| 14 | Mamberamo River Basin Forest Carbon Project | Conservation International (CI) | 1 |
| 15 | Cyclops Mountains REDD Pilot Project | Flora and Fauna International (FFI) | 1 |
| 16 | Jayapura REDD+ Readiness | World Wide Fund for Nature (WWF) | 1 |
| 17 | Sustainable Management of Poigar Forest | Office National des Forêts (ONF) International | 3 |
| 18 | Sulbar Habitat REDD+ | Keepthehabitat | 1 |
| 19 | Berau Forest Carbon Project | The Nature Conservency (TNC) | 1 |
| 20 | FORCLIME - Berau Demonstration Activity (REDD via SFM strengthening) | GoI, Berau District Gov, GFA, GTZ-KfW | 2 |
| 21 | FORCLIME - Malinau Demonstration Activity (Kayan Mentarang NP) | GoI, Berau District Gov, GFA, GTZ-KfW | 2 |
| 22 | East Borneo Project | Global Green | 3 |
| 23 | Hutan Lestari untuk Orangutan: Kehje Sewen Forest | Borneo Orangutan Survival(BOS) | 1 |
| 24 | REDD in Kutai Barat District (i-REDD project) | World Wide Fund for Nature (WWF) | 1 |
| 25 | Kalimantan Forest and Climate Partnership (KFCP) | GoA, GoI, Care | 2 |
| 26 | Katingan Peat Conservation Project | Starling Resources, PT RMU | 3 |
| 27 | Community Carbon Project for Lamandau Wildlife Reserve | RARE & YAYORIN | 1 |
| 28 | REDD in Sebangau National Park | World Wide Fund for Nature (WWF) | 1 |
| 28 | Rewetting of Peatland to avoid emission in Sebangau National Park | World Wide Fund for Nature (WWF) | 1 |
| 29 | The Rimba Raya Biodiversity Reserve Project | Infinite Earth / OFI | 3 |
| 30 | Danau Siawan-Belida Ecological Restoration Concession | FFI & PT. Wana Hijau Nusantara | 1 |
| 31 | FORCLIME - Kapuas Hulu Demonstration Activity (Sustainable Management and Conservation of Peat swamp forest) | GoI, Berau District Gov, GFA, GTZ-KfW | 2 |
| 32 | Ketapang Community Carbon Pool (KCCP): Laman Satong & Permatang Gadung Village | Flora and Fauna International (FFI) | 1 |
| 33 | Kapuas Hulu Community Carbon Pool: Piasak & Jongkong Kiri Hilir Villages & Nanga Betung Village | Flora and Fauna International (FFI) | 1 |
| 34 | Reducing Emission from Deforestation caused by the Oil Palm Sector in Ketapang: PT. Cipta Usaha Sejati & PT. Jalin Vaneo | Flora and Fauna International (FFI) | 1 |
| 35 | Reducing Emission from Deforestation caused by the Oil Palm Sector in Ketapang: PT. Kayong Agro Lestari | Flora and Fauna International (FFI) | 1 |
| 36 | Korea-Indonesia Joint Project for Adaptation and Mitigation of Climate Change in Forestry ( North Batu kliang protection forest ) | Forest Research and Development Agency (FORDA) | 2 |

Figure S 5: Distance between REDD+ projects (centroid) and their nearest PA (boundary)

Table S 7: Forest Area covered by REDD+ projects according to forest categories.

| **Forest Category** | **No. of cells** | **AREA (ha)** | **% of REDD+ area** |
| --- | --- | --- | --- |
| Mangrove | 15 | 37,500 | 0.3 |
| Peat Swamp Forest | 453 | 1,132,500 | 8.9 |
| Lowland forest | 2263 | 5,657,500 | 44.2 |
| Lower Montane forest | 806 | 2,015,000 | 15.8 |
| Upper Montane forest | 372 | 930,000 | 7.3 |
| Plantation / regrowth | 1207 | 3,017,500 | 23.6 |

Appendix S 7: Summary statistics for carbon and biodiversity distribution


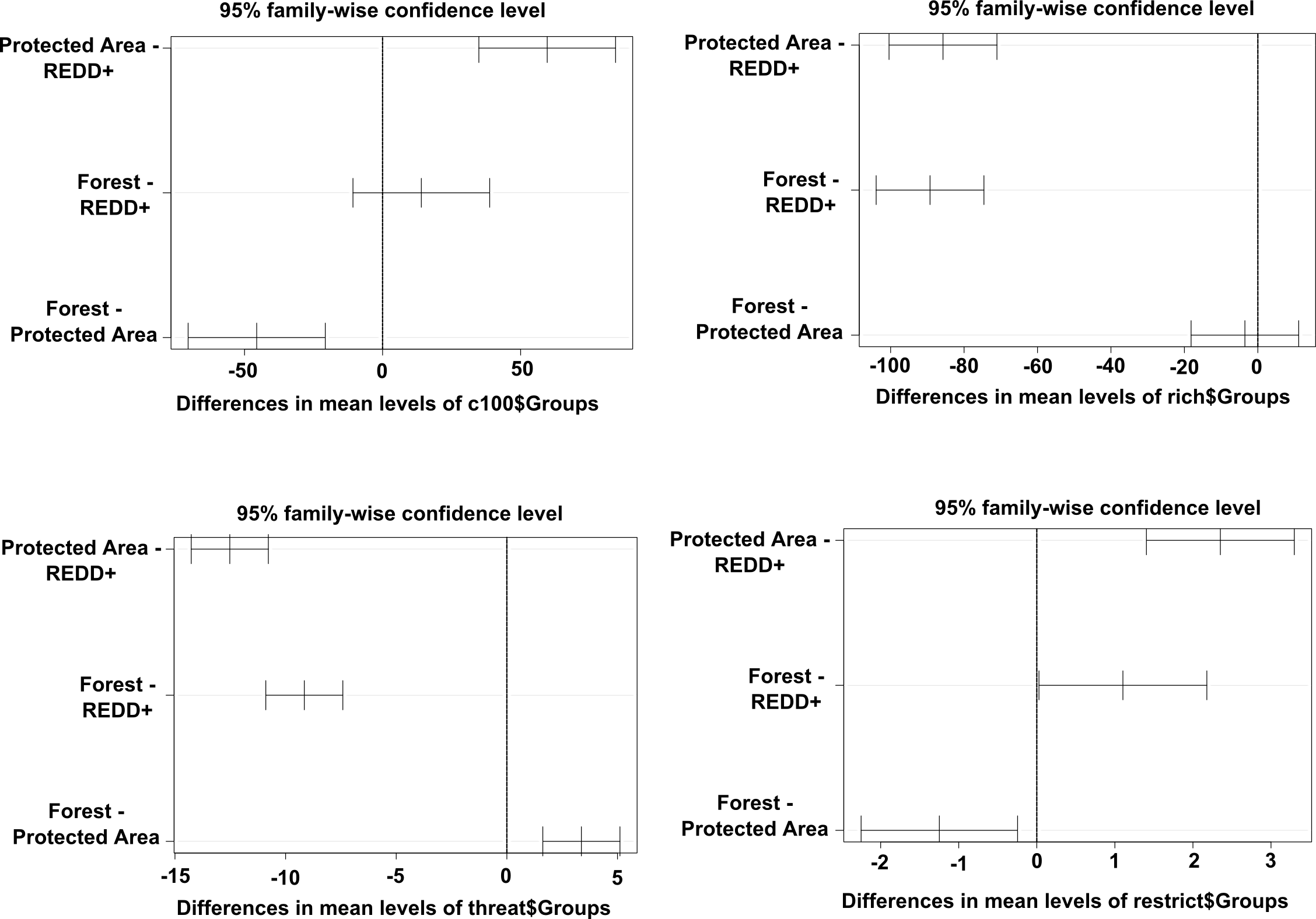


**D**

**C**

**B**

**A**

Figure S 6: TukeyHSD test results showing difference between means between REDD, PA and non-protected forest for the distribution of carbon (A) and three measures of vertebrate richness - total species richness (B), threatened species richness (C) and restricted range species richness (D)


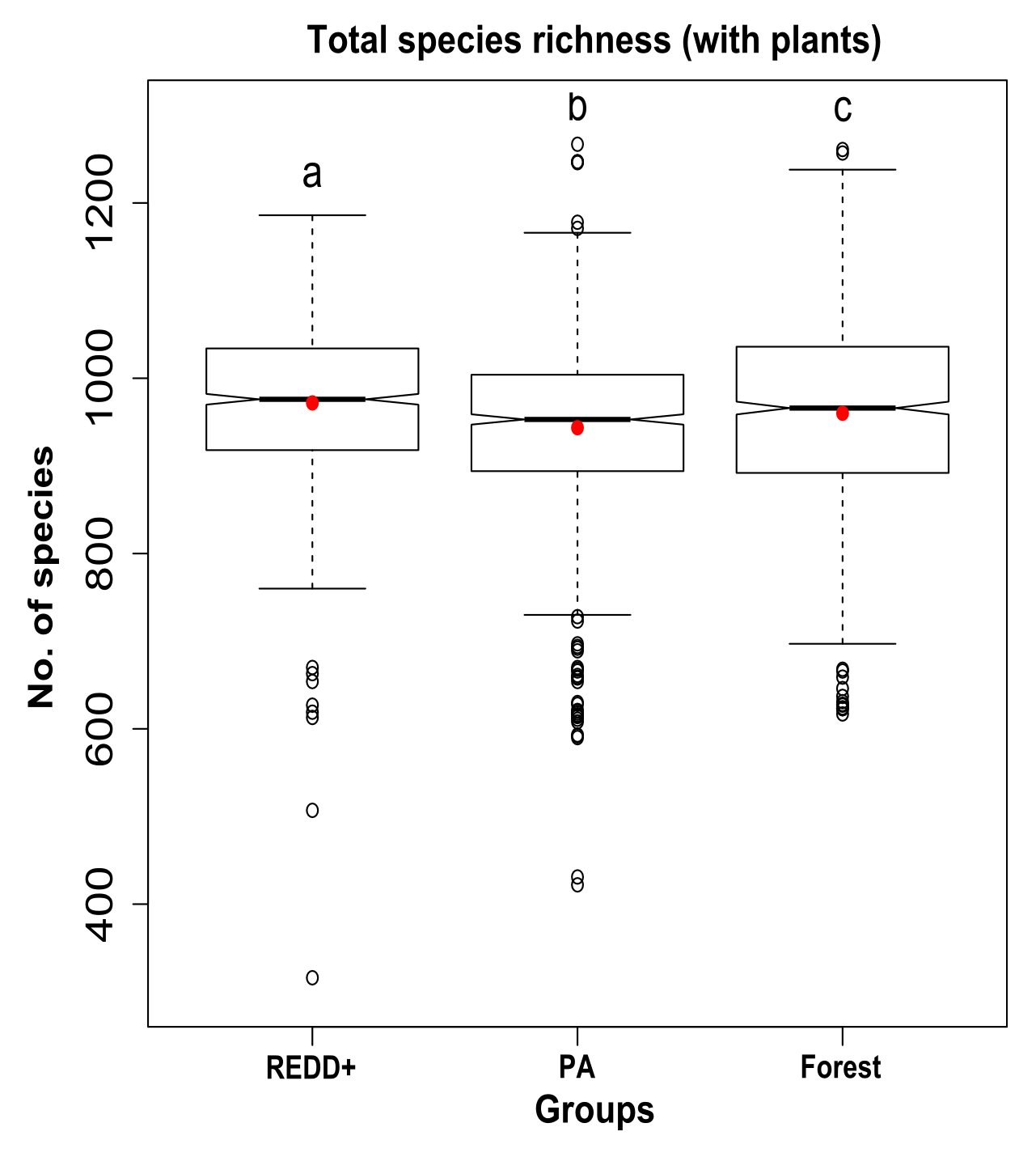

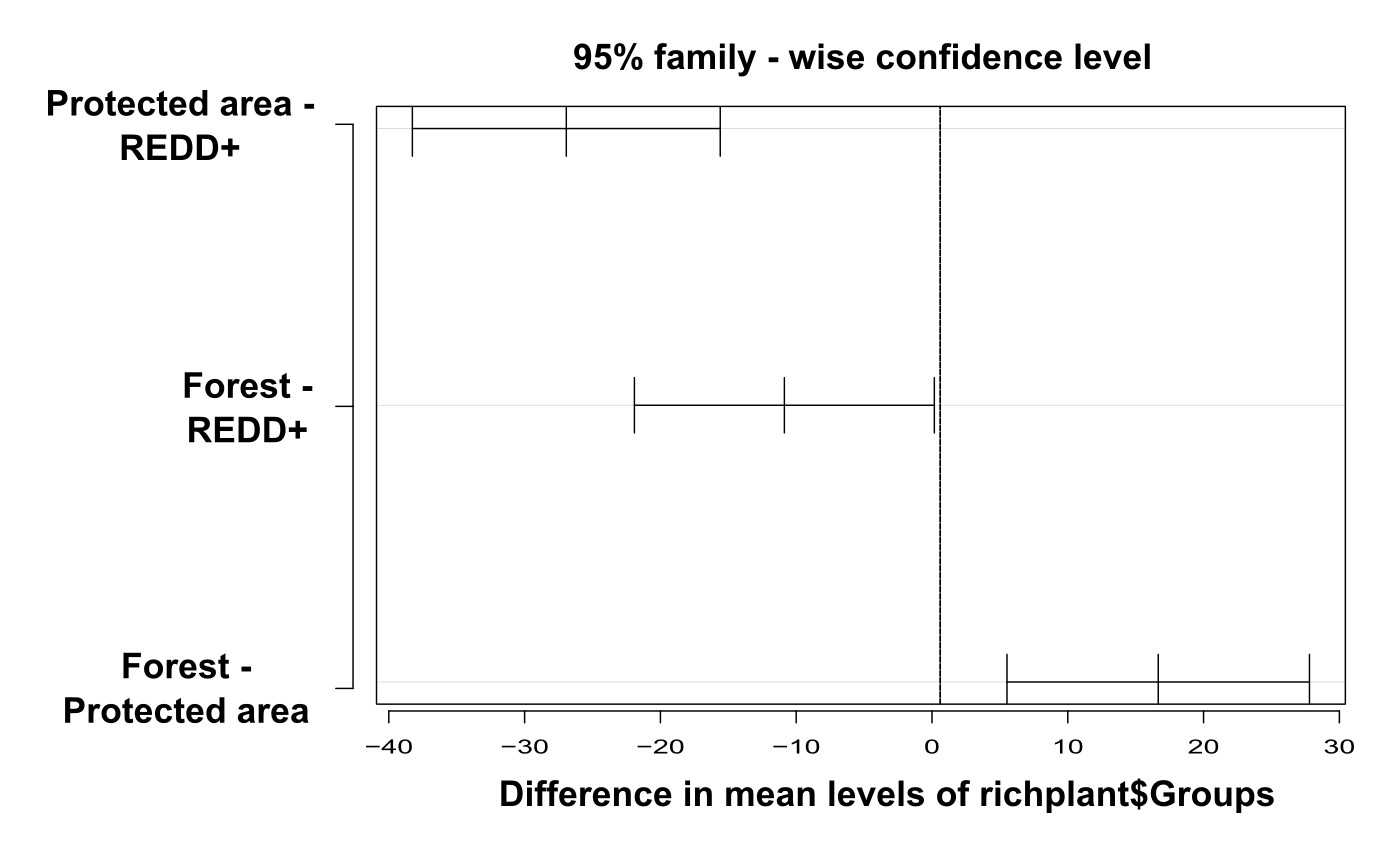


Figure S 7: Results for total species richness with the inclusion of plants a proxy for biodiversity. With the inclusion of plants, REDD+ projects still have on average higher overall species richness per cell (mean = 960) compared to PAs (mean= 917) and un-protected forests (mean = 943). The TukeyHSD test results show that the means of all three groups are significantly different.

Table S 8: Summary statistics for the ANOVA and post hoc TukeyHSD test on 1000 random sampled points from three groups (REDD+ project| PA| Forest) for carbon and biodiversity

| **Carbon Density** | | | | | |
| --- | --- | --- | --- | --- | --- |
| **Analysis of Variance (ANOVA)** | | |  |  |  |
|  | Df | Sum Sq | Mean Sq | F Value | Pr(>F) |
| Carbon$ Group | 2 | 1865721 | 932860 | 17.39 | 3.1e-08 *** |
| Residuals | 2877 | 154296834 | 53631 |  |  |
| **Tukey HSD : Multiple comparisons of means at 95% family-wise confidence level** | | | | | |
|  | diff | Lower | Upper | p adj |  |
| REDD - PA | 59.63312 | 34.87191 | 84.39433 | 0.000000 |  |
| Forest - REDD | 14.03688 | -10.72433 | 38.79809 | 0.378951 |  |
| Forest - PA | -45.59623 | -70.43454 | -20.75792 | 0.000051 |  |
| **Total Species Richness** | | | | | |
| **Analysis of Variance (ANOVA)** | | |  |  |  |
|  | Df | Sum Sq | Mean Sq | F Value | Pr(>F) |
| Rich $ Group | 2 | 5043416 | 2521708 | 130.2 | <2e-16 *** |
| Residuals | 2966 | 57456250 | 19372 |  |  |
| **Tukey HSD : Multiple comparisons of means at 95% family-wise confidence level** | | | | | |
|  | diff | Lower | Upper | p adj |  |
| REDD - PA | -85.736388 | -100.42775 | -71.04502 | 0.000000 |  |
| Forest - REDD | -89.261184 | -103.93414 | -74.58823 | 0.000000 |  |
| Forest - PA | -3.524796 | -18.17538 | 11.12578 | 0.839168 |  |
| **Threatened Species Richness** | | | | | |
| **Analysis of Variance (ANOVA)** | | |  |  |  |
|  | Df | Sum Sq | Mean Sq | F Value | Pr(>F) |
| Threat $ Group | 2 | 82151 | 41076 | 152.2 | <2e-16 *** |
| Residuals | 2930 | 790668 | 270 |  |  |
| **Tukey HSD : Multiple comparisons of means at 95% family-wise confidence level** | | | | | |
|  | diff | Lower | Upper | p adj |  |
| REDD - PA | -12.521847 | -14.264234 | -10.779461 | 0.000000 |  |
| Forest - REDD | -9.154111 | -10.895157 | -7.413064 | 0.000000 |  |
| Forest - PA | 3.367736 | 1.624461 | 5.111011 | 0.000018 |  |
| **Restricted Range Species Richness** | | | | | |
| **Analysis of Variance (ANOVA)** | | |  |  |  |
|  | Df | Sum Sq | Mean Sq | F Value | Pr(>F) |
| Restrict $ Group | 2 | 1654 | 827.1 | 17.2 | 4.07e-08 *** |
| Residuals | 1631 | 78450 | 48.1 |  |  |
| **Tukey HSD : Multiple comparisons of means at 95% family-wise confidence level** | | | | | |
|  | diff | Lower | Upper | p adj |  |
| REDD - PA | 2.351206 | 1.40413412 | 3.2982784 | 0.000000 |  |
| Forest - REDD | 1.10306 | 0.02794969 | 2.1781705 | 0.042785 |  |
| Forest - PA | -1.248146 | -2.2504065 | -0.2458858 | 0.009883 |  |
| **Total species richness (+plants)** | | | | | |
| **Analysis of Variance (ANOVA)** | | |  |  |  |
|  | Df | Sum Sq | Mean Sq | F Value | Pr(>F) |
| Rich $ Group | 2 | 355954 | 177977 | 16.35 | 8.77e-08 *** |
| Residuals | 2730 | 29724232 | 10888 |  |  |
| **Tukey HSD : Multiple comparisons of means at 95% family-wise confidence level** | | | | | |
|  | diff | Lower | Upper | p adj |  |
| REDD - PA | -28.27771 | -39.921213 | -16.634201 | 0.000000 |  |
| Forest - REDD | -11.78453 | -23.127052 | -0.4420015 | 0.039521 |  |
| Forest - PA | 16.49318 | 5.052993 | 27.933367 | 0.002116 |  |
|  |  |  |  |  |  |
| Note: Signif. codes: 0 ‘***’ 0.001 ‘**’ 0.01 ‘*’ 0.05 ‘.’ 0.1 ‘ ’ 1 | | | | |  |

Appendix S 8: Additional information on the deforestation m

We predict deforestation in the absence (reference scenario) and with carbon payments using the econometric model OSIRIS-Indonesia v1.5 for the observed percent deforestation from 2000 to 2005 Busch *et al.*, (2010). We modelled a deforestation scenario “without REDD+ incentives” in order to assess the location of proposed REDD+ projects (in relation to PAs and other non-protected forests) and how threatened they are to future deforestation threat. Observable site characteristics used to proxy for costs included slope, elevation, distance to the nearest road, distance to the nearest provincial capital and the percentage of cells contained within a national park, other protected area, logging concession, timber concession, or estate crop concession (Busch *et al.* 2012) .

**Predicted deforestation without REDD+:** Deforestation was predicted at every 5km x 5km cell in the absence of carbon payment.

The model showed a -21.1% reduction in deforestation due to REDD+ incentives in all forest types throughout Indonesia, with the biggest changes in peatland; with deforestation rates of 1.86% / ha (without REDD+) and 1.15% / ha (with REDD+). We categorized deforestation threat in Indonesian into 5 categories (very low – very high) using Natural Breaks to determine the classes. We then extracted deforestation rates under both scenarios for REDD+ projects, PAs and other non-protected forests and assess the amount of areas which fell within each threat categories.

Figure S6 shows the distribution of deforestation threat in Indonesia under in the absence of REDD+ incentives and with REDD+ incentives as well as the location of REDD+ projects and PAs in Indonesia.

**REFERENCES**

BirdLife International and NatureServe. 2012. Bird species distribution maps of the world. BirdLife International, Cambridge, UK and NatureServe, Arlington, USA. Available from http://www.birdlife.org/datazone/info/spcdownload.

Busch, J., R. Lubowski, F. Godoy, D. Juhn, K. Austin, J. Hewson, and M. Steininger. 2010. Open Source Impacts of REDD+ Incentives Spreadsheet – Indonesia (OSIRIS-Indonesia). http://sp10.conservation.org/osiris/Pages/overview.aspx.

Busch, J., R. N. Lubowski, F. Godoy, M. Steininger, A. a Yusuf, K. Austin, J. Hewson, D. Juhn, M. Farid, and F. Boltz. 2012. Structuring economic incentives to reduce emissions from deforestation within Indonesia. Proceedings of the National Academy of Sciences of the United States of America **109**:1062–7.

Gaston, K. J., and R. a. Fuller. 2009. The sizes of species’ geographic ranges. Journal of Applied Ecology **46**:1–9.

Hurlbert, A. H., and W. Jetz. 2007. Species richness, hotspots, and the scale dependence of range maps in ecology and conservation. Proceedings of the National Academy of Sciences of the United States of America **104**:13384–9.

IUCN. 2012. Global mammal assessment. Available from http://www.iucnredlist.org/technical-documents/spatial-data#mammals.

IUCN. 2013. Global amphibian and reptile assessment. Available from http://www.iucnredlist.org/technical-documents/spatial-data#amphibians.

Jenkins, C. N., S. L. Pimm, and L. N. Joppa. 2013. Global patterns of terrestrial vertebrate diversity and conservation. Proceedings of the National Academy of Sciences of the United States of America **110**:E2602–10.

Mardiastuti, A. 2012. The role of UN-REDD in the development of REDD+in Indonesia Volume III: highlights of REDD+ related projects in Indonesia. UN-REDD Programme Indonesia.

Miettinen, J., C. Shi, W. J. Tan, and S. C. Liew. 2012. 2010 land cover map of insular Southeast Asia in 250-m spatial resolution. Remote Sensing Letters **3**:11–20.

Paoli, G. D. et al. 2010. Biodiversity Conservation in the REDD. Carbon balance and management **5**:7.

Possingham, H. P., S. J. Andelman, M. A. Burgman, A. Rodrigo, L. L. Master, and D. A. Keith. 2002. Limits To The Use Of Threatened Species Lists **17**:503–507.

Pusat Standardisasi dan Lingkungan. 2011. Prosiding Workshop Review status Pilot REDD+ di Indonesia, Jakarta 21 Desember 2010. Page 240. UN-REDD Programme Indonesia, Jakarta.

Raes, N., M. C. Roos, J. W. F. Slik, E. E. Van Loon, and H. Ter Steege. 2009. Botanical richness and endemicity patterns of Borneo derived from species distribution models. Ecography **32**:180–192.

Raes, N., L. G. Saw, P. C. van Welzen, and T. Yahara. 2013. Legume diversity as indicator for botanical diversity on Sundaland, South East Asia. South African Journal of Botany **89**:265–272.

Ramdani, F., and M. Hino. 2013. Land Use Changes and GHG Emissions from Tropical Forest Conversion by Oil Palm Plantations in Riau Province, Indonesia. PLoS ONE **8**.

Rocchini, D., J. Hortal, S. Lengyel, J. M. Lobo, a. Jimenez-Valverde, C. Ricotta, G. Bacaro, and a. Chiarucci. 2011. Accounting for uncertainty when mapping species distributions: The need for maps of ignorance. Progress in Physical Geography **35**:211–226.

1. Sundaland or the Sunda shelf which includes Borneo, Sumatra, Java, Bali and the Peninsular Malaysia [↑](#footnote-ref-1)
2. http://www.redd-indonesia.org/, http://www.forestsclimatechange.org/, http://www.forestcarbonasia.org, http://forestclimatecenter.org, http://redd-database.iges.or.jp/redd/, http://www.theredddesk.org/countries, http://www.climate-standards.org/category/projects/, http://www.gcftaskforce-database.org/. [↑](#footnote-ref-2)
